# ChatGPT, large language models, and artificial intelligence in medicine and health care: A primer for clinicians and researchers

**DOI:** 10.1016/j.jdin.2023.07.011

**Published:** 2023-07-29

**Authors:** Jonathan Kantor

**Affiliations:** Department of Dermatology, Center for Global Health, and Center for Clinical Epidemiology and Biostatistics, Perelman School of Medicine, University of Pennsylvania, Philadelphia, Pennsylvania; Florida Center for Dermatology, St Augustine, Florida; and Alchemy Labs, Oxford, UK

**Keywords:** AI, artificial intelligence, annotation, ChatGPT, Google Bard, large language models, medical education, Microsoft Bing, practice management

Generative language models, such as OpenAI’s ChatGPT, Google Bard, Microsoft Bing Chat, and others, have in the span of just a few months, revolutionized the ways in which physicians, researchers, and the general public interact with the corpus of knowledge available on the internet. By providing a natural language interface, these models facilitate information retrieval and amalgamation, allowing interactive conversations that provide immediate—if not always accurate^1^—answers in real time.

Generative language models offer promise in patient education, medical education, medical office management, research, and clinical care. For patient education, they allow physicians and staff to quickly generate tailored informational handouts with (sometimes) pitch-perfect prose, while in medical education, these systems can serve as virtual tutors for trainees and experienced clinicians alike. In medical office management, generative language models can assist in automating administrative tasks, such as appointment scheduling, patient triage, and responding to frequently asked questions. Indeed, generating standardized scripts for office staff (after a thorough vetting by the physician) is a particularly easy to implement and time-saving application of this technology. In research, these models have the potential to analyze vast amounts of medical literature, aiding in literature reviews, data extraction, and hypothesis generation, although the promise of these approaches should be tempered by the potentially devastating risk of such models yielding frankly incorrect or even counterfactual results.^2^ In clinical care, such models promise to better generate (hopefully) meaningful clinic notes in a fraction of the time while also allowing physicians to quickly ask clinical questions and solve medical conundrums. The promise, therefore, is that large language models could allow us to level the playing field; they will not replace master clinicians, but they may make middling ones a bit less middling.

Yet there are also risks in adopting generative language models in health care. First, although these systems are trained on vast amounts of data, these data are not always current. ChatGPT has a cutoff date of September 2021; it is largely ignorant of anything that has transpired over the past 2 years. In medicine, as in many other fields, this can be a lifetime, although future iterations will likely address such limitations. Second, ethical issues have been raised regarding the tendency of large langue models to not only reflect but also amplify bias.^3^^,^^4^ Although various fixes for this have been rolled out, many (perhaps ironically) require human oversight, and they remain less than perfect. Third, such models may hallucinate or confabulate, and this shortcoming is only aggravated by automation bias (the tendency we have to ascribe more gravitas than deserved to results of computer-generated efforts), a problem likely underscored by the slickly confident tone and grammatically on-point construction of large language model output. Still, despite these shortcomings large language models are not only here to stay but—particularly as they become integrated within existing health care information technology systems—are likely to take on an outsized role in medicine in the future. Ultimately, as with the annotation underlying so much of today’s artificial intelligence boon, it will take a human touch to decide how to best leverage these models while mitigating risk.

## Conflicts of interest

None disclosed.

## References

[1] O’Hern K., Yang E., Vidal N.Y. (2023). ChatGPT underperforms in triaging appropriate use of Mohs surgery for cutaneous neoplasms. JAAD Int.

[2] Salvagno M., Taccone F.S., Gerli A.G. (2023). Can artificial intelligence help for scientific writing?. Crit Care.

[3] Beltrami E.J., Grant-Kels J.M. (2023). Consulting ChatGPT: ethical dilemmas in language model artificial intelligence. J Am Acad Dermatol.

[4] Verhoeven F., Wendling D., Prati C. (2023). ChatGPT: when artificial intelligence replaces the rheumatologist in medical writing. Ann Rheum Dis.

